# S(+)-ibuprofen destabilizes MYC/MYCN and AKT, increases p53 expression, and induces unfolded protein response and favorable phenotype in neuroblastoma cell lines

**DOI:** 10.3892/ijo.2013.2148

**Published:** 2013-10-25

**Authors:** NAOHIKO IKEGAKI, SAKEENAH L. HICKS, PAUL L. REGAN, JOSHUA JACOBS, AMINA S. JUMBO, PAYTON LEONHARDT, ERIC F. RAPPAPORT, XAO X. TANG

**Affiliations:** 1 Department of Anatomy and Cell Biology, College of Medicine, University of Illinois at Chicago, Chicago, IL;; 2 Nucleic Acid PCR Core, The Children’s Hospital of Philadelphia, Philadelphia, PA, USA

**Keywords:** neuroblastoma, MYCN, MYC, p53, favorable neuroblastoma genes

## Abstract

Neuroblastoma is a common pediatric solid tumor that exhibits a striking clinical bipolarity favorable and unfavorable. The survival rate of children with unfavorable neuroblastoma remains low among all childhood cancers. MYCN and MYC play a crucial role in determining the malignancy of unfavorable neuroblastomas, whereas high-level expression of the favorable neuroblastoma genes is associated with a good disease outcome and confers growth suppression of neuroblastoma cells. A small fraction of neuroblastomas harbors *TP53* mutations at diagnosis, but a higher proportion of the relapse cases acquire *TP53* mutations. In this study, we investigated the effect of S(+)-ibuprofen on neuroblastoma cell lines, focusing on the expression of the MYCN, MYC, AKT, p53 proteins and the favorable neuroblastoma genes *in vitro* as biomarkers of malignancy. Treatment of neuroblastoma cell lines with S(+)-ibuprofen resulted in a significant growth suppression. This growth effect was accompanied by a marked decrease in the expression of MYC, MYCN, AKT and an increase in p53 expression in neuroblastoma cell lines without *TP53* mutation. In addition, S(+)-ibuprofen enhanced the expression of some favorable neuroblastoma genes (*EPHB6, CD44*) and genes involved in growth suppression and differentiation (*EGR1, EPHA2, NRG1* and *SEL1L*). Gene expression profile and Ingenuity pathway analyses using *TP53-*mutated SKNAS cells further revealed that S(+)-ibuprofen suppressed molecular pathways associated with cell growth and conversely enhanced those of cell cycle arrest and the unfolded protein response. Collectively, these results suggest that S(+)-ibuprofen or its related compounds may have the potential for therapeutic and/or palliative use for unfavorable neuroblastoma.

## Introduction

Neuroblastoma is a neural crest-derived tumor that is the most common extracranial pediatric malignancy. The tumor accounts for 7–10% of all childhood cancers and is the cause of ∼15% of cancer deaths in children. The clinical behavior of neuroblastoma is highly variable. Nearly 50% of the tumors exhibit very aggressive behavior. These tumors are classified as high-risk neuroblastoma and are characterized by widespread tumor dissemination, late relapse and poor long-term survival (1). The other half of tumors is treatable by surgical resection and/or low dose chemotherapy. In some cases, the patients are simply observed without a therapeutic intervention. We have shown that high-level expression of certain genes, termed favorable neuroblastoma genes, is associated with the favorable subset of patients with a good disease outcome (2). In addition, when ectopically expressed in unfavorable neuroblastoma cell lines, the favorable neuroblastoma genes confer growth suppression. *MYCN* amplification is detected in ∼20% of all neuroblastoma cases and is significantly associated with advanced stage disease, rapid tumor progression and shorter survival (3). Notably, neuroblastoma was the first human malignant tumor in which amplification of a proto-oncogene was found in primary tumor specimens (4).

Cyclooxygenases (COXs) are thought to play an important role in the regulation of progression, invasiveness and angiogenesis of various cancers (5). In fact, Johnsen *et al*, showed that primary neuroblastoma specimens and cell lines expressed varying levels of COX-2 and that the growth of neuroblastoma cell lines *in vitro* and xenografts was suppressed when COX inhibitors were administered (6). However, the response of neuroblastoma cell lines to COX inhibitors did not appear to correlate with levels of COX-2 expressed in these cells. In addition, the downstream effector of COXs, prostaglandin E(2), has been implicated in playing a role in neuroblastoma cell differentiation by promoting the production of cAMP (7). These observations suggest that the effect of COX inhibitors on neuroblastoma cells may not solely be due to inhibition of COXs in the cells.

Our previous study on the response of neuroblastoma cells to inhibitors of histone deacetylases and proteasomes suggests that enhanced p53 expression is linked to MYCN destabilization (8). We also showed that inhibition of Hsp90 resulted in the destabilization of AKT, MYC, MYCN and in an increase in p53 levels (9). In this study, we continued our effort to identify small molecules that can destabilize or downregulate MYC and MYCN protein expression in neuroblastoma cells. We have found that S(+)-ibuprofen destabilizes MYC and MYCN proteins in five well-characterized neuroblastoma cell lines. This effect of S(+)-ibuprofen was accompanied by the augmented expression of p53 and by the reduction in AKT expression. These findings are similar to our previous report on the effect of Hsp90 inhibition in neuroblastoma cell lines (9). In addition, treatment of neuroblastoma cell lines with S(+)-ibuprofen resulted in an enhanced expression of favorable neuroblastoma genes and genes associated with growth suppression. Moreover, gene expression profiling and Ingenuity pathway analysis on *TP53*-mutated SKNAS cells have shown that S(+)-ibuprofen suppresses growth-promoting pathways and conversely enhances pathways that are growth suppressive. S(+)-ibuprofen also augmented many genes related to the unfolded protein response (UPR) (10). Collectively, this study suggests that S(+)-ibuprofen can reduce the malignancy of unfavorable neuroblastoma cells by modulating the expression of MYC, MYCN, AKT, p53 and by changing the gene expression profile towards a less aggressive phenotype. A possible link between MYC/MYCN destabilization and UPR in S(+)-ibuprofen treated neuroblastoma cells is also discussed.

## Materials and methods

### Neuroblastoma cell lines

The neuroblastoma cell lines were grown in RPMI-1640 supplemented with 5% (v/v) fetal bovine serum and OPI (1 mM oxaloacetate, 0.45 mM pyruvate, 0.2 U/ml insulin, at final concentrations). These cell lines tested negative for mycoplasma and their identity was validated by the original source or by microsatellite analysis (P.S. White, Children’s Hospital of Philadelphia, unpublished data). IMR5 (a clone of IMR32) and CHP134 were received from Dr Roger H. Kennett (Wheaton College, Wheaton, IL, USA). SY5Y and SKNBE(2)C were from Robert Ross (Fordham University). SKNAS was from Dr C. Patrick Reynolds (The Texas Tech University Health Sciences Center, Lubblock, TX, USA). CHP134, IMR5 and SKNBE(2)C are MYCN-amplified cell lines, whereas SY5Y and SKNAS are non-MYCN-amplified cell lines. In addition, SKNBE(2)C and SKNAS are known to harbor TP53 mutations (11,12).

### MTS assay

An MTS (3-(4,5-dimethylthiazol-2-yl)-5-(3-carboxymethoxyphenyl)-2-(4-sulfophenyl)-2H-tetrazolium, inner salt) assay (a water soluble form of the MTT assay) was performed as described in our previous study (2). S(+)-ibuprofen was purchased from LKT Laboratories (St. Paul, MN, USA). The stock solution was made at 0.5 M as sodium salt in H_2_O, filter-sterilized and stored at −20°C.

### Western blot analysis

Western blotting was performed as previously described (8,9). Light emission signals were captured by a LAS-3000 (Fujifilm) digital image analyzer. Cell extracts were made in 2D gel sample buffer (9 M urea, 2% Nonidet-P40, 2% 2-mercaptoethanol and 0.32% of pH 3.0–10.0 2D Pharmalyte) and the protein content of the samples was determined by the Bio-Rad protein assay kit using bovine serum albumin as a standard and the sample buffer as the blank. Antibodies used to detect proteins of interest are described in the figure legends.

### Reverse transcription and TaqMan real-time PCR

RNAs were isolated from neuroblastoma cell lines using the Qiagen RNeasy kit. Total RNA (2 μg) was used to synthesize cDNA. The experimental procedures for the reverse transcription were performed as previously described (13). The quantitative real-time PCR was done using an iQ5 real-time PCR machine (Bio-Rad). TaqMan probes were purchased from Applied Biosystems, Inc. and the multiplex qPCR mix (QuantiTect Multiplex PCR kit) was purchased from Qiagen. Relative quantification of expression levels of genes of interest was done by the ΔΔCt method (14) using the expression of 18S ribosomal RNA as an internal control. The experimental procedures were performed according to the instructions provided by Qiagen and Bio-Rad.

### Affymetrix microarray analysis

SKNAS cells were treated with 1 mM S(+)-ibuprofen for 3 days. The S(+)-ibuprofen-treated or non-treated control cells were harvested and subjected to gene expression profiling analysis. Total RNAs were isolated from the control or treated cells using an RNeasy Kit (Qiagen). cRNA targets were prepared from 1 *μ*g of total RNA using the MessageAmp Premier III kit from Ambion. Fragmented target (15 *μ*g) was hybridized to U133 plus V2 chips at 45°C at 60 rpm for 16 h and the chips were washed according to the manufacturer’s instructions. Chips were scanned and the data were collected using the Affymetrix Command Console software. The microarray hybridizations were performed in triplicate both for the untreated control and the S(+)-ibuprofen-treated cells. CEL files generated by Command Console were used for subsequent data extraction and analysis using Partek Genomics Suite (v. 6.6). Data were extracted using GCRMA (15) and differentially expressed genes determined using ANOVA under default Partek settings. The gene list for pathway analysis was generated using a 2-fold cutoff and a 0.05 p-value adjusted for false discovery rate (FDR).

### Ingenuity pathway analysis

Ingenuity pathway analysis (IPA) was used to build knowledge-based networks (Ingenuity Systems, Redwood City, CA, USA). Matrices were generated with fold-change and p-value for each of the comparisons. Identifiers for the Affymetrix probe sets were uploaded into IPA and these were mapped to the corresponding genes. These genes were overlaid onto the global molecular network developed from information contained in the Ingenuity Pathways Knowledge Base. Networks of the genes in the data set were then algorithmically generated based on their connectivity. These networks were scored based on the number of molecules included in the networks, with the score corresponding to the negative logarithm of the p-value corresponding to the presence of these molecules in the network by chance.

## Results

### The growth suppressive effect of S(+)-ibuprofen and its MYCN/MYC destabilization in neuroblastoma cell lines

**Figure 1.**
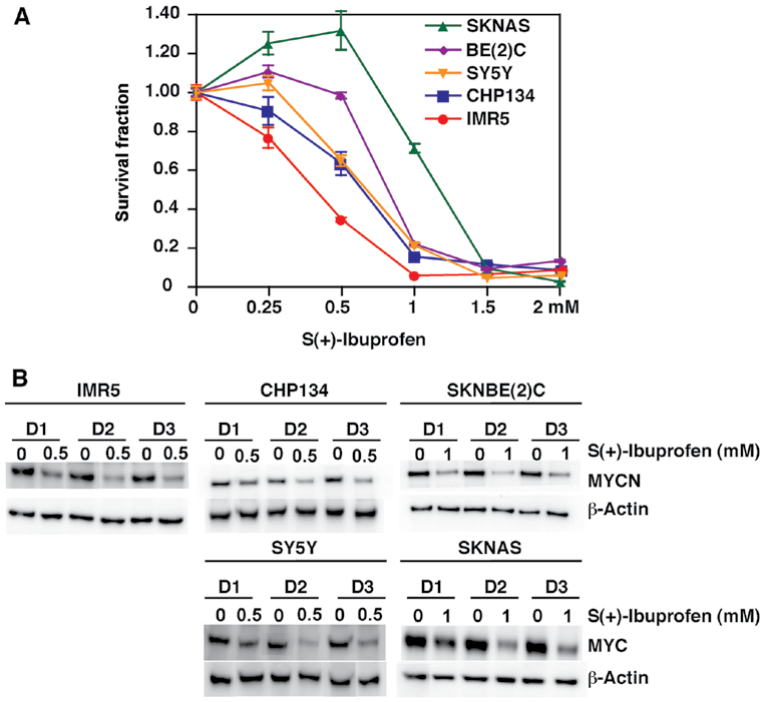
(A) S(+)-ibuprofen treatment results in significant growth suppression of neuroblastoma cell lines. Neuroblastoma cells were treated with S(+)-ibuprofen at the concentrations indicated. Two days after the treatments, an MTS assay was done to determine the effect of the drug on growth of the neuroblastoma cell lines indicated. (B) Treatment of neuroblastoma cells with S(+)-ibuprofen results in a decrease in MYCN expression [IMR5, CHP134, SKNBE(2)C] and MYC expression [SY5Y and SKNAS]. IMR5, CHP134, SKNBE(2)C, SY5Y and SKNAS were treated with S(+)-ibuprofen as indicated for one, two and three days. The cells were harvested and subjected to western blot analysis. Total protein (5 *μ*g) was loaded per lane. The MYCN-specific monoclonal antibody, NCM II 100, was used to detect expression of MYCN (33). The MYC-specific monoclonal antibody, 9E10 (ATCC) and anti-β-Actin monoclonal antibody (C4, Santa Cruz Biotechnology) were used to detect expression of MYC and β-Actin, respectively.

It has been reported that non-steroidal anti-inflammatory drugs (NSAIDs) (Diclofenac and Celecoxib) can induce apoptosis and inhibit neuroblastoma growth (6). However, the underlying mechanism of the anti-growth effect of these drugs in neuroblastoma has not been investigated (6). In this study, we tested the growth suppressive effect of S(+)-ibuprofen, which is also an NSAID, in neuroblastoma cells and investigated the effect of S(+)-ibuprofen on MYCN/MYC expression in these cells. *MYCN*-amplified cell lines [IMR5, CHP134, SKNBE(2) C] and non-*MYCN*-amplified cell lines [SY5Y and SKNAS] were treated with S(+)-ibuprofen at different doses as indicated for two days and MTS assay was performed to assess the growth effect of S(+)-ibuprofen. As shown in Fig. 1A, the neuroblastoma cell lines tested showed different susceptibility to S(+)-ibuprofen: IMR5 was the most sensitive and SKNAS was the most resistant. There was no correlation between the S(+)-ibuprofen susceptibility and the status of *MYCN* amplification. However, SKNBE(2)C and SKNAS were the two most resistant cell lines to S(+)-ibuprofen and are known to harbor *TP53* mutations (11,12). Fig. 1B shows the results of the effect of S(+)-ibuprofen on MYCN and MYC stability in neuroblastoma cell lines. Control untreated IMR5, CHP134 and SKNBE(2)C cells express high levels of MYCN, whereas the untreated-SY5Y and SKNAS cells express high levels of MYC. As shown in Fig. 1B, S(+)-ibuprofen at 0.5 mM [IMR5, CHP134, SY5Y] and at 1.0 mM [SKNBE(2)C and SKNAS] decreased MYCN or MYC expression, respectively in a time-dependent manner. The MYC/MYCN destabilizing effect of S(+)-ibuprofen was seen as early as day one of the drug treatment.

### Treatment of neuroblastoma cells with S(+)-ibuprofen results in an increased p53 expression

**Figure 2.**
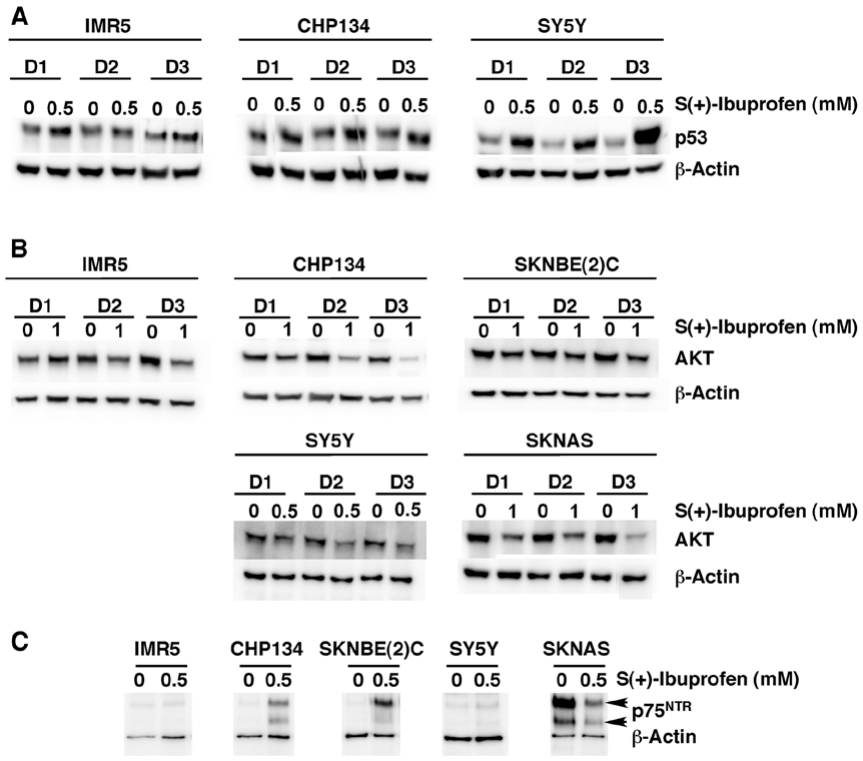
(A) Treatment of neuroblastoma cells with S(+)-ibuprofen results in an increase in p53 expression in IMR5, CHP134 and SY5Y. IMR5, CHP134 and SY5Y were treated with S(+)-ibuprofen (0.5 mM) for one, two and three days. The cells were harvested and subjected to western blot analysis. Total protein (5 *μ*g) was loaded per lane. A p53-specific monoclonal antibody, DO-1 (Calbiochem), was used to detect expression of p53. (B) The effect of S(+)-ibuprofen on AKT expression in neuroblastoma cells. IMR5, CHP134, SKNBE(2)C, SY5Y and SKNAS were treated with S(+)-ibuprofen at the doses indicated for one, two and three days. With the exception of SY5Y, expression of AKT was not decreased in the other four cell lines when they were treated with S(+)-ibuprofen at 0.5 mM (data not shown). The cells were harvested and subjected to western blot analysis. Total protein (5 *μ*g) was loaded per lane. An anti-AKT rabbit polyclonal antibody (Cell Signaling) was used to detect the expression of AKT protein. (C) The expression of p75^NTR^ in response to S(+)-ibuprofen treatment in neuroblastoma cell lines. IMR5, CHP134, SKNBE(2)C, SY5Y and SKNAS were treated with S(+)-ibuprofen (0.5 mM) for three days. The cells were harvested and subjected to western blot analysis. Total protein (5 *μ*g) was loaded per lane. The rabbit monoclonal anti-p75^NTR^ clone D4B3 (Cell Signaling) was used to detect p75^NTR^. The appearance of p75^NTR^ as doublets on western blot assay is due to its glycosylation.

Our previous study indicated that elevated p53 expression has a suppressive effect on MYCN expression in *MYCN*-amplified neuroblastoma cells (8). We thus investigated whether this was also the case in the S(+)-ibuprofen-treated cells. The SKNBE(2)C and SKNAS cell lines were not included in this experiment because these cell lines harbor *TP53* mutations (11,12). As shown in Fig. 2A, S(+)-ibuprofen at the concentration of 0.5 mM increased p53 expression in all cell lines in a time-dependent manner.

### S(+)-ibuprofen downregulates AKT expression in neuroblastoma cells

The effect of S(+)-ibuprofen on MYC/MYCN and p53 in neuroblastoma cell lines was similar to that of the inhibition of Hsp90 in neuroblastoma cells as shown in our earlier study (9). In addition, we previously reported that the Hsp90 inhibition was accompanied by downregulation of AKT in neuroblastoma cells. We therefore examined if S(+)-ibuprofen had any effect on AKT expression in neuroblastoma cells. As shown in Fig. 2B, S(+)-ibuprofen at the concentrations of 0.5–1 mM suppressed the expression of AKT in a time-dependent manner.

### The expression of p75^NTR^ did not correlate with the growth suppressive effect of S(+)-ibuprofen in neuroblastoma cell lines

In bladder and prostate cancer cell lines, ibuprofen and its related compounds were shown to cause upregulation of p75^NTR^ and the growth suppression. In addition, the survival inhibiting effects of the drugs were attributed to the enhanced expression of p75^NTR^ independent of COX inhibition (16,17). To investigate if this is the case for neuroblastoma cells, the expression of p75^NTR^ was examined in five neuroblastoma cell lines treated with S(+)-ibuprofen. As shown in Fig. 2C, there was no correlation between the expression of p75^NTR^ and the growth suppressive effect of S(+)-ibuprofen (Fig. 1A) in neuroblastoma cell lines.

### The effect of S(+)-ibuprofen on favorable neuroblastoma genes (EPHB6, CD44) and growth suppressive genes (EGR1, NRG1, SEL1L, EPHA2) in neuroblastoma cells

**Figure 3.**
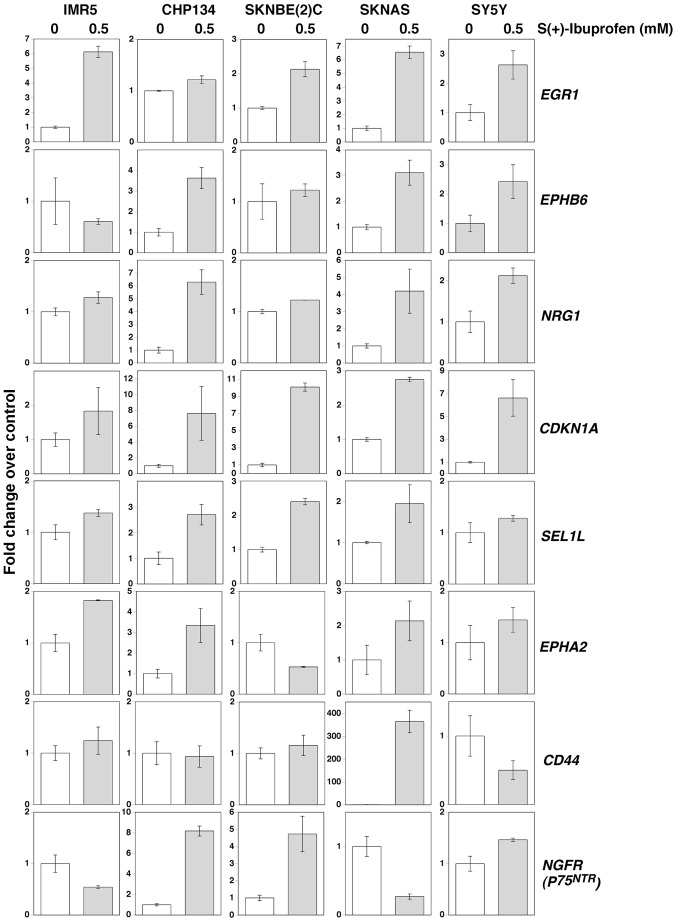
Enhanced expression of favorable neuroblastoma genes (*EPHB6* and *CD44*) and growth suppressive genes (*CDKN1A, EGR1, SEL1L, NRG1, EPHA2* and *NGFR* or *p75^NTR^*) in S(+)-ibuprofen-treated neuroblastoma cell lines. IMR5, CHP134, SKNBE(2)C, SKNAS and SY5Y cells were treated with S(+)-ibuprofen as indicated for three days. RNAs were prepared from the control untreated cells and the drug-treated cells. Expression of genes of interest was examined in duplicate by TaqMan real-time PCR using gene specific TaqMan Gene Expression assays (ABI). Expression levels of the genes of interest are presented as fold change in the S(+)-ibuprofen-treated cells over the untreated control.

In order to assess if S(+)-ibuprofen could suppress the malignancy of neuroblastoma cells by utilizing growth suppression pathways, we examined the expression of favorable neuroblastoma genes and genes associated with growth suppression of neuroblastoma cells. As shown in Fig. 3, among the favorable neuroblastoma genes examined (*EPHB6, EFNB2, EFNB3, TrkA, CD44, MIZ-1*), S(+)-ibuprofen augmented the expression of *EPHB6* significantly in four of five neuroblastoma cell lines tested. The expression of *CD44* was augmented only in SKNAS. In addition, the expression of several genes associated with growth suppression, including *CDKN1A, EGR1, NRG1* and *SEL1L* (9), were also enhanced in all S(+)-ibuprofen-treated neuroblastoma cell lines. The expression of the neuroblastoma growth suppressive gene, *EPHA2* (18) was significantly increased in four of five neuroblastoma cell lines.

### Global effect on gene expression mediated by treatment of S(+)-ibuprofen in SKNAS cells

To gain further insight into mechanisms by which S(+)-ibuprofen suppresses aggressive neuroblastoma cell growth, we performed gene expression profiling and Ingenuity pathway analysis using SKNAS cells. SKNAS is a *TP53*-mutated neuroblastoma cell line, expresses high levels of MYC and is highly tumorigenic in mouse xenograft assays (19). In spite of these malignant features, our data showed that S(+)-ibuprofen exhibited its efficacy against SKNAS cells. To explore additional mechanisms of S(+)-ibuprofen action on malignant neuroblastoma cells, SKNAS cells were treated with S(+)-ibuprofen at 1 mM for three days and gene expression profiling was done and compared to that of the control untreated cells. Affymetrix U133 plus V2 chips represent >47,000 transcripts. Of those, we found 754 genes were upregulated and 1008 genes were downregulated in S(+)-ibuprofen-treated SKNAS cells compared to the non-treated control cells, using the threshold values of >2-fold change and FDR-adjusted p-value of <0.05.

**Figure 4.**
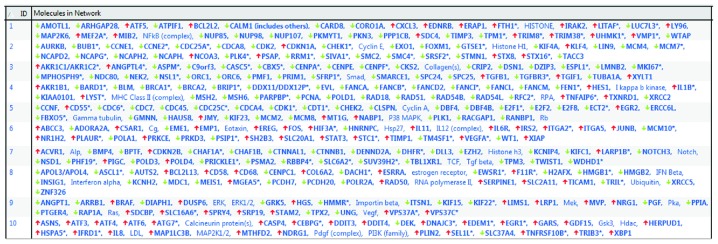
Genes in the top ten networks that are affected by the S(+)-ibuprofen treatment in SKNAS cells. Gene expression profiles of control and S(+)-ibuprofen treated SKNAS were analyzed by the Ingenuity pathway analysis program. The top ten networks that were affected by S(+)-ibuprofen treatment and genes included in the networks are shown. The arrows (up and down) also indicate the directional changes in gene expression.

Ingenuity pathway analysis (IPA) was next performed to identify correlations between the genes significantly changed in expression and the functional networks in the IPA database. The functional networks identified were then ranked by score. Table I shows the scores of the top 10 networks affected by the S(+)-ibuprofen treatment in SKNAS cells along with the top functions associated with each. The biological functions include cell cycle, cellular assembly and organization, DNA replication, recombination and repair, cellular growth and proliferation, cell death and survival, cell signaling, cardiovascular system development and function, hereditary disorder, embryonic development, gene expression, cell morphology, cellular movement, cellular function and maintenance and cellular compromise. Fig. 4 shows the genes included in the top 10 Functional networks and the directional response (up or down) to the S(+)-ibuprofen treatment.

**Figure 5.**
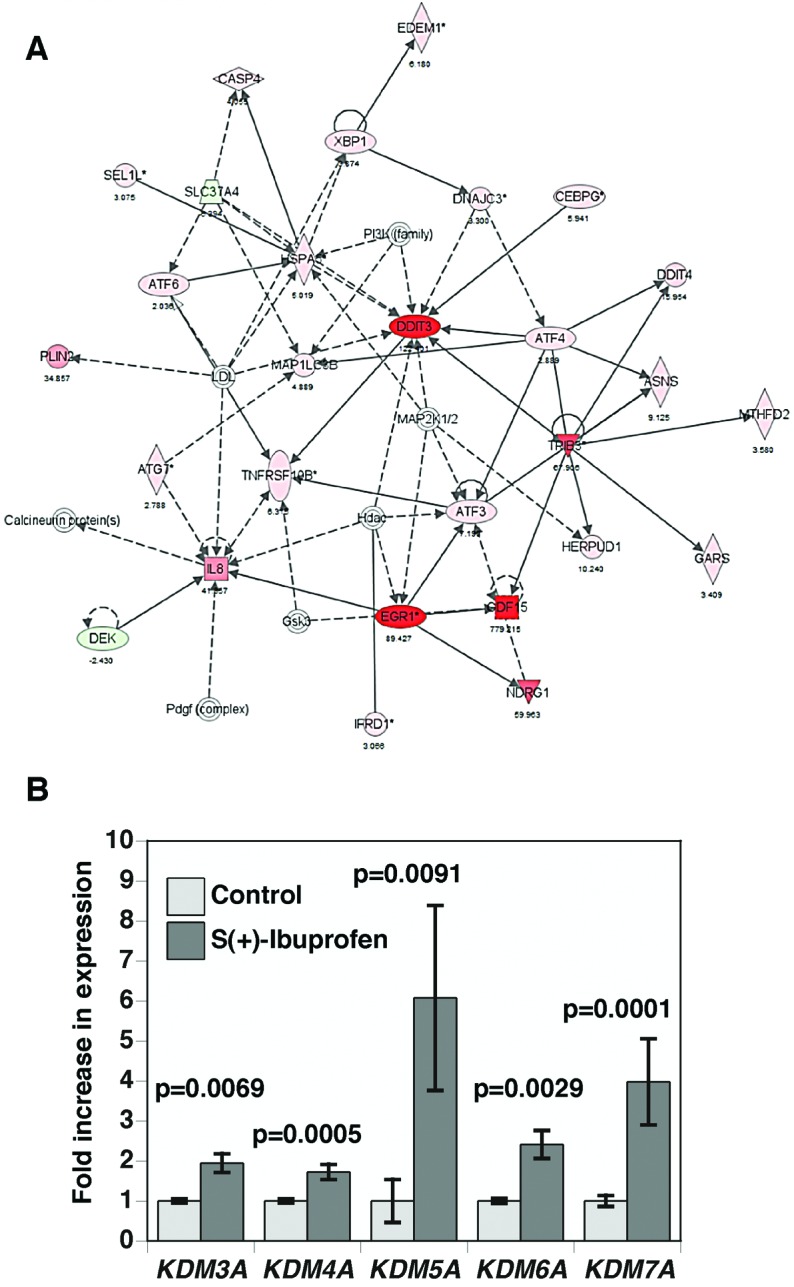
(A) Interaction among the genes in Network 10. *GDF15, DDIT3* and *EGR1* are among the six most upregulated genes in response to S(+)-ibuprofen treatment in SKNAS cells. Many genes included in this network are known to be associated with UPR. (B) Increase in the expression of transcripts encoding histone demethylases in response to S(+)-ibuprofen treatment in SKNAS cells. Normalized hybridization intensities of the genes indicated on microarrays were plotted. For each gene, there are at least three data points.

As shown in Fig. 4, many genes involved in cell growth, cell cycle and DNA replication were downregulated in the S(+)-ibuprofen-treated SKNAS cells, including *MKI67, PCNA, CCNE1, CCNE2, CDK1, CDK2, CDC6, CDC7, CHEK1, CHEK2, FOXM1, MCM2, MCM4, MCM7, MCM8, MSH2, MSH6, AURKB, BUB1, PLK4, E2F1, and E2F2*. Conversely, several genes involved in growth suppression and cell cycle arrest were upregulated by S(+)-ibuprofen in SKNAS cells, including *CDKN1A, CDKN2B, EGR1, and SEL1L*. Some of the stem cell and mesenchymal signature genes, *EZH2, NOTCH3, DLL3* and *TWIST1* were also downregulated by S(+)-ibuprofen. In addition, genes related to the UPR were upregulated by S(+)-ibuprofen, including *ATF3, ATF4, ATF5, ATF6, DDIT3, DDIT4, DNAJC3, EDEME1, HSPA5, GDF15, HERPUD1, NDRG1, and XBP1*. As shown Fig. 5A, *GDF15, DDIT3* and *EGR1* are among the six most upregulated genes in response to S(+)-ibuprofen treatment in SKNAS cells. Moreover, *KDM3A, KDM4A, KDM5A, KDM6B* and *KDM7A/JHDM1D*, encoding histone demethylases, were upregulated in S(+)-ibuprofen-treated SKNAS cells (Fig. 5B).

## Discussion

S(+)-ibuprofen, also known as Dexibuprofen, is the active stereoisomer of ibuprofen racemate and it is shown to be equally efficacious to Diclofenac as an NSAID (20). It has been shown that neuroblastoma cell lines and primary tumor specimens express COX-2 and that COX inhibitors, Diclofenac and Celecoxib exhibit a growth suppressive effect on neuroblastoma cells. As mentioned above, several observations suggest that the growth suppressive effect of COX inhibitors in neuroblastoma involves other mechanisms than the inhibition of COX enzymes.

MYCN and MYC play a crucial role in determining the malignancy of unfavorable neuroblastomas (3,21–23), whereas high-level expression of favorable neuroblastoma genes is associated with a good disease outcome and confers growth suppression of neuroblastoma cells (24). Results of this study have shown that S(+)-ibuprofen destabilized MYCN/MYC in neuroblastoma cell lines with or without *MYCN* amplification. S(+)-ibuprofen also destabilized AKT, a key enzyme to regulate cell survival (25,26), in neuroblastoma cells. In addition, S(+)-ibuprofen enhanced the expression of p53 in neuroblastoma cell lines with normal p53 function. Taken together, these results suggest that S(+)-ibuprofen can reduce the malignant phenotype of the neuroblastoma cell lines though multiple mechanisms.

We have also shown that S(+)-ibuprofen was growth suppressive to neuroblastoma cell lines with a *TP53* mutation, though the efficacy was less compared to those with no *TP53* mutation. Gene expression profiling and Ingenuity pathway analyses using the *TP53* mutated-SKNAS cell line reveals that S(+)-ibuprofen suppresses biological pathways associated with cell growth/cell cycle progression and conversely augments pathways associated with cell cycle arrest and the UPR. Notably, the expression of several genes associated with UPR found in human B cells (10) was augmented significantly in S(+)-ibuprofen-treated SKNAS cells compared to the non-treated control cells.

UPR is known to induce conformational changes and oligomerization of the proapoptotic proteins Bax and Bak localized on the ER membrane. These changes lead to the creation of pores in the ER membrane, allowing Ca^2+^ mobilization from ER to the cytosol (27,28). Consequently, Ca^2+^ released from the ER activate calpains. The activated calpains then activate caspase 12 to mediate apoptosis. Calpain has also been shown to cleave MYC family proteins, giving rise to MYC fragments, called MYC-Nicks (29,30). Although calpain cleavage of Myc is constitutive, the cleavage is enhanced under conditions that require rapid downregulation of Myc levels (29,30). One of the mechanisms by which MYC and MYCN are destabilized in S(+)-ibuprofen-treated neuroblastoma cells may be due to the augmented activation of calpains in response to UPR.

It has been reported that some NSAIDs induce the UPR or ER stress response in gastric mucosal cells, leading to death of the cells *in vitro* (31). It is thus possible that one of the biological pathways to exhibit the growth suppressive effect of S(+)-ibuprofen on neuroblastoma cells includes the UPR. It is also found that several genes encoding histone demethylases (*KDM3A, KDM4A, KDM5A, KDM6B* and *KDM7A/JHDM1D*) were upregulated in S(+)-ibuprofen-treated SKNAS cells. It is of interest to note that among these genes, *KDM6B* is also an ATF4 target gene (32). S(+)-ibuprofen can thus change the epigenetic status of the cells and modulates transcriptional activity. It remains to be seen whether the UPR itself has any direct effect on the epigenetic status of the cells.

Collectively, this study suggests that S(+)-ibuprofen can reduce the malignancy of unfavorable neuroblastoma cells by modulating the expression of MYC, MYCN, AKT and p53, by changing the gene expression profile towards a less aggressive phenotype and by inducing UPR, which may link to destabilization of MYC and MYCN. Hence, S(+)-ibuprofen or its related compounds may have the potential for therapeutic and/or palliative use for unfavorable neuroblastoma.

## Figures and Tables

**Table I.** Biological functions associated with top 10 networks affected by S(+)-ibuprofen treatment in SKNAS cells.

Networks	Score	Focus molecules	Top functions
1	33	33	Cell signaling, cellular assembly and organization, DNA replication, recombination and repair
2	33	33	Cell cycle, cellular assembly and organization, DNA replication, recombination and repair
3	33	33	Cell cycle, cellular assembly and organization, DNA replication, recombination and repair
4	31	32	Cell cycle, DNA replication, recombination and repair, developmental disorder
5	29	31	Cell cycle, DNA replication, recombination and repair, cellular assembly and organization
6	29	31	Cellular growth and proliferation, cell death and survival, cardiovascular system development and function
7	27	30	Developmental disorder, hereditary disorder, embryonic development
8	27	30	Gene expression, cell cycle, DNA replication, recombination and repair
9	24	28	Cellular movement, cell signaling, cell cycle
10	24	28	Cell morphology, cellular function and maintenance, cellular compromise

## Abbreviations:

UPR: unfolded protein response
